# Supplementary material for: WordCommentsAnalyzer: A windows software tool for qualitative research
Source: F1000Res. 2018 Sep 4;7:536. Originally published 2018 May 3. [Version 2] doi: 10.12688/f1000research.14819.2 (PMC6137411; doi:10.12688/f1000research.14819.2)
Supplement: Supplementary file 3 [file f1000research-7-17614-s0002.tgz › b2a79496-4ada-47c4-9682-a99ce867836d.zip!/Eight Iranian Health Journals/Archives of Rehabilitation_36.docx]

© Archives of Rehabilitation. This work is licensed under CC BY-NC 4.0 (https://creativecommons.org/licenses/by-nc/4.0/).

Volume & Issue: دوره 11 - شماره 4

Title: بررسی مهارتهای ادراک بینایی در کودکان عادی ۷ تا ۱۳ ساله شهر تهران

http://rehabilitationj.uswr.ac.ir/article-1-762-fa.html

هدف: این مطالعه با هدف بررسی مهارتهای ادراک بینایی در کودکان عادی ۷ تا ۱۳ ساله شهر تهران و تعیین تأثیر جنسیت، سن و موقعیتهای اجتماعی- تحصیلی مختلف بر آن انجام شد. روش‌بررسی: در این مطالعه تحلیلی و مقطعی، از بین دانش‌آموزان ۷ تا ۱۳ ساله کلیه مدارس مناطق مختلف آموزش و پرورش شهر تهران، به روش خوشه‌ای پنج منطقه و چهل مدرسه ابتدایی و راهنمایی انتخاب و متناسب با جمعیت آنها، ۷۸۸ نفر به‌صورت تصادفی ساده انتخاب و در سه گروه سنی طبقه‌بندی شده و توسط آزمون مهارتهای ادراک بینایی– تجدید نظر شده (تی.وی.پی.اس-آر) مورد ارزیابی قرار گرفتند. داده‌های حاصل با استفاده از آزمونهای کولموگروف- اسمیرنوف، بارتلت، آنالیز واریانس، آزمون تی مستقل، کروسکال والیس، یومن‌ویتنی و ضریب همبستگی اسپیرمن مورد تجزیه و تحلیل قرار گرفت. یافته‌ها: نمرات استاندارد در کلیه مهارتهای ادراک بینایی در حد متوسط بود. بین گروههای سنی، بجز در مورد حافظه بینایی و حافظه توالی بینایی، در بقیه مهارتها اختلافی معنادار وجود داشت (مقدار احتمال از 0/045 تا کمتر از 0/100). بین دانش‌آموزان مناطق مختلف آموزش و پرورش، در تمام مهارتها، اختلاف معنادار وجود داشت (P<0/001) . بین کودکان دختر و پسر در ثبات شکل بینایی(۰۰۹/۰=پی)، تشخیص شکل از زمینه (P<0/001) و بهره ادراک بینایی (P=0/011) اختلاف معنادار وجود داشت، درحالی‌که در سایر مهارتها، بین آنها تفاوت معنادار مشاهده نشد. همبستگی بین خرده‌آزمونها با کل آزمون (Rho>0/617)، از همبستگی بین خرده‌آزمونها با یکدیگر (Rho<0/508) کمتر بود. نتیجه‌گیری: برخلاف انتظار، افزایش سن کودکان با ادراک بینایی ارتباط معنادار، ولی معکوس داشت. جنسیت و موقعیت اجتماعی - شهری منطقه آموزش و پرورش نیز در برخی از خرده‌آزمونهای مهارتهای ادراک بینایی موجب تفاوت معنادار بین کودکان عادی شد. ضمن این مطالعه، برای هریک از مهارتها نمره هنجار به‌دست آمد که می‌تواند به‌عنوان معیاری در مطالعات بعدی مورد استفاده قرار گیرد.

Title: بررسی تأثیر یک برنامه درمانی تلفیقی در بیماران دچار نشانگان درد ناحیه‌ای مزمن نوع یک

http://rehabilitationj.uswr.ac.ir/article-1-763-fa.html

هدف: نشانگان درد ناحیه ‌ ای مزمن یکی از مهمترین و شاید بدخیم ‌ ترین اشکال مشکلات عصبی به ‌ خصوص در اندام فوقانی می ‌ باشد. تعیین تأثیر توانبخشی با تلفیق روشهای فیزیوتراپی و کاردرمانی در درمان نشانگان درد ناحیه ‌ ای مزمن هدف این مطالعه بود.   روش بررسی: در این مطالعه شبه ‌ تجربی که از نوع قبل و بعد می ‌ باشد، ۲۰ بیمار مبتلا به نشانگان درد ناحیه ‌ ای مزمن (سی . آر . پی . اس . ) نوع یک با نمونه ‌ گیری ساده انتخاب و میزان درد، دامنه حرکتی، ادم و قدرت عضلانی آنها ثبت شد. سپس مداخلات درمانی که ترکیبی از مدالیته ‌ های فیزیوتراپی و کاردرمانی بود، طی ۲۰ جلسه و یک روز در میان توسط یک گروه درمانگر انجام شد. پس از دوره درمانی بیماران مجدد اً بررسی شدند. داده ‌ ها با استفاده از آزمون آماری تی زوجی تحلیل شد.   یافته‌ها: پس از درمان مشاهده شد که میانگین میزان درد و ادم بیماران به ‌ طور معنا ‌ داری کاهش و دامنه حرکتی و قدرت گیرش به ‌ طور معناداری افزایش یافت (P>0/05). نتیجه‌گیری: اجرای به ‌ موقع و تلفیق روشهای فیزیوتراپی و کاردرمانی همزمان در بیماران مبتلا به نشانگان درد ناحیه ‌ ای مزمن نوع اول موجب کاهش درد و تورم و افزایش دامنه حرکتی مفاصل و قدرت گرفتن دست می ‌ شود.

Title: تحول نظریه ذهن دانش‌آموزان عقب‌مانده ذهنی و ارتباط آن با تعداد خواهران و برادران

http://rehabilitationj.uswr.ac.ir/article-1-283-fa.html

هدف: این پژوهش با هدف بررسی تحول نظریه ذهن دانش‌آموزان عقب‌مانده ذهنی و رابطه آن با تعداد خواهران و برادران انجام شد. روش‌بررسی: در این پژوهش توصیفی که از نوع تعیین همبستگی است، تمامی دانش‌آموزان پسر عقب‌مانده ذهنی ۸ تا ۱۴ سال (۵۹ نفر) که در سال تحصیلی ۸۸-۸۷ در پایه‌های اول تا پنجم دبستان در مرکز آموزشی میعاد شهر تربت‌حیدریه (تنها مرکز آموزشی پسرانه) مشغول به تحصیل بودند، شرکت داشتند. برای جمع‌آوری داده‌های مربوط به نظریه ذهن از تکلیف جعبه گول‌زننده (محتوای غیرمنتظره) و آزمون نظریه ذهن ۳۸ سئوالی استفاده شد. داده‌های بدست آمده با آزمونهای آماری تحلیل واریانس یک طرفه، شفه، همبستگی پیرسون و مجذور کای اسکوئر تجزیه و تحلیل شدند. یافته‌ها: تحول نظریه ذهن سطح اول و دوم دانش‌آموزان عقب‌مانده ذهنی تا ۱۲ سالگی صعودی (P<0/001) و پس از آن ثابت است (P=0/87). ولی تحول نظریه ذهن که با تکلیف محتوای غیرمنتظره سنجیده می‌شود، صعودی است (P=0/02). همچنین تعداد خواهران و برادران با تحول نظریه ذهن دانش‌آموزان عقب‌مانده ذهنی رابطه معناداری ندارد (P=0/52). نتیجه‌گیری: تحول نظریه ذهن دانش‌آموزان عقب‌مانده ذهنی بسته به اینکه چه نوع تکلیفی برای سنجش نظریه ذهن استفاده شود، متفاوت است. در مجموع ادعای رویکرد «نظریه نظریه» مبنی بر اینکه تحول نظریه ذهن براساس بلوغ فرایندهای ضروری صورت می‌گیرد، مورد پذیرش است. ادعای نظریاتی که بر پایه نظریه‌های اجتماعی ـ فرهنگی هستند، مبنی بر اینکه تجربه با سایر افراد به عنوان منشاء رشد درک ذهن است، باید بیشتر مورد بررسی قرار گیرد.

Title: بررسی تأثیر آزمون مستقیم بالا آوردن پا به‌صورت فعال بر تونوسیته و فعالیت عضلات ثبات‌دهنده لگن در زنان

http://rehabilitationj.uswr.ac.ir/article-1-244-fa.html

هدف: آزمون مستقیم بالا آوردن پا (اس.ال.آر.) به‌صورت فعال در تشخیص اختلال عملکردی مفصل خاجی- خاصره‌ای به‌عنوان یک آزمون معتبر تشخیصی، کیفیت انتقال نیرو بین تنه و پا را بررسی می‌کند. هدف این مطالعه، مقایسه تغییرات تون و مقدار فعالیت عضلات ثبات‌دهنده لگن در حین انجام آزمون مستقیم بالا آوردن پا به‌صورت فعال بین زنان سالم و مبتلا به درد مفصل خاجی- خاصره‌ای می‌باشد. روش بررسی: این مطالعه مقایسه‌ای، به‌صورت مورد- شاهدی، بر روی 11 زن مبتلا به درد مفصل خاجی- خاصره‌ای و 15 زن سالم که همگی در محدوده سنی 19 تا 50 سال قرار داشته و به‌صورت ساده انتخاب شدند، انجام شد. الکترومیوگرافی سطحی از عضلات مستقیم شکمی، مایل خارجی، مایل داخلی، نزدیک‌کننده طویل، دوسر رانی، سرینی بزرگ و راست‌کننده ستون مهره‌ها در دو وضعیت استراحت و حین آزمون فعال به‌عمل آمده و تونوسیته عضلات ثبت شد. داده‌های تحقیق با استفاده از آزمون تی مستقل مقایسه شد. یافته‌ها: عضله دوسر رانی به‌طور معناداری در گروه بیمار نسبت به گروه سالم از تون استراحت بالاتری برخوردار بود(P<0/05). در گروه مبتلایان به درد مفصل خاجی- خاصره‌ای، میزان فعالیت برخی از عضلات ثبات‌دهنده لگن به‌طور معناداری در حین انجام آزمون اس.ال.آر. فعال کمتر از گروه سالم بود (P<0/05). نتیجه‌گیری: افزایش تون استراحت عضله دوسر رانی و کاهش فعالیت عضلات ثبات‌دهنده لگن حین آزمون مستقیم بالا آوردن پا به‌صورت فعال در بیماران مبتلا به درد مفصل خاجی- خاصره‌ای، نمایانگر تغییر استراتژی کنترل حرکت در ثبات ناحیه کمری- لگنی می‌باشد. این مسئله می‌تواند در انتقال نیرو از خلال لگن اختلال ایجاد نماید.

Title: استانداردسازی مقیاس رشدی-‌حرکتی پی‌بادی در کودکان 60 تا 71 ماهه شهر شیراز

http://rehabilitationj.uswr.ac.ir/article-1-766-fa.html

هدف: ارزیابی کیفیت مهارتهای حرکتی کودکان خردسال با استفاده از ابزاری استاندارد، راهکار تشخیصی مهمی برای کشف ناتوانایی ‌ های حرکتی و تأخیر رشدی می ‌ باشد. از این رو این مطالعه با هدف استاندارد کردن مقیاس رشدی - ‌ حرکتی پی ‌ بادی جهت بررسی مهارتهای حرکتی کودکان خردسال انجام شد. روش بررسی: در این مطالعه توصیفی و اعتبارسنجی 150 کودک عادی دختر و پسر با دامنه سنی 60 تا 71 ماه، به ‌ روش نمونه ‌ برداری تصادفی خوشه ‌ ای چند- ‌ مرحله ‌ ای از بین مهدکودکهای موجود در 6 منطقه شهری شهرستان شیراز انتخاب و مهارتهای حرکتی درشت و ظریف آنها با آزمون پی ‌ بادی مورد بررسی قرار گرفت. ابتدا پایایی و روایی آزمون بررسی شد و سپس به ‌ منظور استاندارد کردن آن مطابق توانایی ‌ های حرکتی کودکان مورد بررسی، نمرات خام بدست آمده از سنجش عملکرد حرکتی به نمرات استاندارد تبدیل گردید. تحلیل نتایج از طریق تعیین همبستگی پیرسون و محاسبه ضریب همبستگی درون طبقه ‌ ای صورت گرفت. یافته‌ها: ضرایب همبستگی بالا حاکی از روایی و پایایی مناسب آزمون بود، به ‌ طوری ‌ که در مورد پایایی، ضریب همبستگی درون طبقه ‌ ای برای توافق در یک آزمونگر 0/987 در مقیاس حرکتی درشت و 0/971 در مقیاس حرکتی ظریف بدست آمد. تألیف جداول نمرات استاندارد از دیگر یافته ‌ های مهم پژوهش حاضر می ‌ باشد. نتیجه‌گیری: متخصصین و کارشناسان توانبخشی می ‌ توانند از آزمون پی ‌ بادی به ‌ عنوان ابزاری استاندارد و دقیق برای معاینه ویژگیهای حرکتی کودکان 60 تا 71 ماهه استفاده کنند و پس از ارزیابی و جاگذاری نمره خام بدست آمده از آزمون پی ‌ بادی در جداول استاندارد این پژوهش به تشخیص مناسب برسند.

Title: بررسی تأثیر حرکات اصلاحی بر انعطاف‌پذیری و قدرت در دختران نوجوان اسکولیوتیک وضعیتی

http://rehabilitationj.uswr.ac.ir/article-1-778-fa.html

هدف: ‌این پژوهش با هدف تعیین تأثیر یک دوره حرکات اصلاحی ویژه بر روی انعطاف‌پذیری و قدرت در دختران نوجوان اسکولیوتیک انجام گرفت.  روش بررسی: مطالعه شبه ‌ تجربی حاضر یک کارآزمایی بالینی است که بر روی چهل نفر از دختران اسکولیوتیک 12 تا 15 ساله انجام شد. نمونه ‌ ها به ‌ صورت ساده و در دسترس انتخاب شده و به ‌ط ور تصادفی ساده به دو گروه 20 نفری آزمایش و کنترل تخصیص یافتند. روی گروه آزمایش یک دوره حرکات اصلاحی به مدت 30 روز اعمال شد، ولی برای گروه کنترل این حرکات اصلاحی انجام نشد و انعطاف ‌ پذیری و قدرت دختران قبل و بعد از حرکات اصلاحی در دو گروه، مورد ارزیابی قرار گرفت. داده ‌ های حاصل با استفاده از آزمونهای آماری تی‌زوجی و تی‌مستقل مورد تجزیه و تحلیل قرار گرفت.  یافته‌ها: اختلاف معناداری در انعطاف‌پذیری و قدرت قبل و بعد از حرکات اصلاحی در گروه آزمایش وجود داشت (P<0/001)، ولی بین دو گروه بعد از اعمال مداخله اختلاف معناداری در انعطاف‌پذیری و قدرت دیده نشد (P=0/6). نتیجه‌گیری: انجام حرکات اصلاحی موجب بهبود انعطاف‌پذیری و قدرت دختران اسکولیوتیک می‌شود.

Title: رابطه سبکهای مقابله با استرس و خودکارآمدی با رضایت از تجربیات بالینی در دانشجویان کارورز رشته‌های توانبخشی

http://rehabilitationj.uswr.ac.ir/article-1-779-fa.html

هدف: هدف از پژوهش حاضر ارزیابی رابطه سبکهای مقابله با استرس و خودکارآمدی با رضایت دانشجویان کارورز توانبخشی از تجارب بالینی خود به ‌ عنوان یک درمانگر بود. روش بررسی: دراین مطالعه توصیفی و مقطعی، 318 دانشجوی کارورز رشته‌های توانبخشی شامل 140 دانشجوی فیزیوتراپی، 97 دانشجوی کاردرمانی و 81 دانشجوی گفتاردرمانی از دانشکده ‌ های توانبخشی کشور به ‌ صورت نمونه ‌ گیری طبقه ‌ ای-تصادفی انتخاب شدند. دانشجویان کارورز توانبخشی بودند. داده ها با استفاده از پرسشنامه های سبک های مقابله، خودکارآمدی عمومی و رضایت از تجربیات بالینی جمع آوری و با استفاده از محاسبه ضریب همبستگی پیرسون و تحلیل رگرسیون چندگانه تحلیل شد. یافته ها: محاسبه همبستگی بین متغیر ها نشان داد که سبک مقابله ارزیابی مجدد مثبت با رضایت از تجربیات بالینی در دانشجویان هر سه رشته همبستگی مثبت معنی دار دارد (P<0/05). سبک های مقابله جستجوی حمایت اجتماعی و حل مدبرانه مسأله نیز با رضایت از تجربیات شغلی دانشجویان کارورز فیزیوتراپی و کار درمانی همبستگی مثبت معنی دار داشت (P<0/05). همچنین رضایت از تجارب بالینی در دانشجویان کارورز هر سه رشته فیزوتراپی، کار درمانی و گفتار درمانی با خودکارآمدی آنها همبستگی مثبت معنی دار داشت (P<0/001). نتایج تحلیل رگرسیون نشانگر این بود که در هر سه رشته، خودکارآمدی در مرحله اول وارد معادله رگرسیون شده و بیشترین سهم را در پیش بینی واریانس رضایت از تجربیات بالینی دانشجویان دارد.. نتیجه گیری: با توجه به یافته های پژوهش می توان نتیجه گرفت که سبک های مقابله و خودکارآمدی به طور معنی داری قادر به پیش بینی رضایت از تجربیات بالینی در دانشجویان فیزیوتراپی، کار درمانی و گفتار درمانی هستند.

Title: مقایسه تاثیر نظام ارتباطی مبادله تصویری و گفتاردرمانی بر رشد ارتباط کودکان 8-4 ساله درخودمانده

http://rehabilitationj.uswr.ac.ir/article-1-780-fa.html

هدف: مطالعه حاضر به مقایسه تاثیر روشهای رایج گفتاردرمانی و پکس بر رشد مهارتهای ارتباطی کودکان درخودمانده بی کلام 8-4 ساله پرداخت. روش بررسی: در این مطالعه تجربی- مقایسه ای، 10 کودک درخودمانده بی کلام با نمونه گیری آسان و طبق معیارهای ورود و خروج وارد مطالعه شدند. آزمودنی ها بر اساس سن و شاخصه های رشدی نیوشا با یکدیگر همتا و با انتخاب خانواده ها وارد گروههای 5 نفره پکس و گفتاردرمانی شدند. متغیرهای گوش کردن، زبان دریافتی و بیانی، شناخت ، گفتار و ارتباط اجتماعی قبل و بعد از مداخله بوسیله شاخص رشدی نیوشا بررسی شدند. مداخله در هر دو گروه به مدت 3 ماه شامل 3 جلسه 40 دقیقه ای در هفته انجام شد .داده های مطالعه با کمک آزمون مجموع رتبه ها و آزمون رتبه ای- نشانه دار ویلکاکسون مورد تحلیل قرار گرفت. یافته‌ها: هر دو گروه در شاخصه های رشدی پیشرفت داشتند . رشد گروه پکس در گوش کردن،زبان دریافتی، شناخت و ارتباط اجتماعی بیشتر از گروه گفتاردرمانی بود. اما آزمونهای آماری تفاوت معناداری بین دو گروه نشان ندادند و مجموع رتبه ها (T) در حد فاصل ارزشهای بحرانی T1 وT2 قرار داشت . نتیجه‌گیری: پکس برای آموزش ارتباط به کودکان درخودمانده بدون کلام روش موثری است و می تواند در کنار روشهای رایج گفتاردرمانی بعنوان روش آموزشی مکمل مورد استفاده قرار گیرد و بعنوان تسهیل کننده ارتباط قبل از دریافت مداخلات گفتاردرمانی و یا همزمان با آن بکار رود.

Title: ارتباط جایگاه ضایعه مغزی با نوع زبان پریشی در بیماران فارسی زبان مبتلا به سکته مغزی

http://rehabilitationj.uswr.ac.ir/article-1-790-fa.html

هدف: سالهاست که بررسی ضایعات مغزی همراه با نشانگان‌های گوناگون زبان‌پریشی اساس پژوهش‌های مربوط به سازمان‌بندی زبان در مغز را شکل می‌دهد. پژوهش حاضر با هدف سنجش ارتباط میان جایگاه ضایعات مغزی و زبان‌پریشی بروکا و ورنیکه در بیماران فارسی‌زبان تک‌زبانه سکته مغزی انجام شد. روش بررسی: این مطالعه از نوع مطالعات تک‌سیستمی (مطالعه با نمونه کوچک) می‌باشد. از میان 120 بیمار دارای سکته مغزی مراجعه‌کننده به کلینیک‌های رفیده، تبسم، مغز و اعصاب کرج، بیمارستان لقمان حکیم و امام خمینی شهر تهران، تنها 9 بیمار (5 مرد و 4 زن) دارای زبان‌پریشی بروکا و 2 بیمار (2 مرد) دارای زبان‌پریشی ورنیکه واجد شرایط شرکت در پژوهش حاضر بودند. برای تشخیص نوع زبان‌پریشی بیماران از آزمون زبان‌پریشی فارسی (اف.ای.تی.)، زیرآزمون درک دستوری نسخه فارسی آزمون زبان‌پریشی دوزبانگی (بی.ای.تی.)، آزمون زبان‌پریشی نامیدن فارسی و ارزیابی کنش‌پریشی (ضمیمه آزمون نامیدن) استفاده شد. تشخیص جایگاه ضایعات مغزی بیماران نیز از طریق تصاویر ام.آر.آی. انجام شد. یافته‌ها: در بیماران زبان‌پریش بروکا (12/51±46 ساله)، نواحی کپسول خارجی – اینسولا، آپرکولوم رولاندیک، شکنج پیشانی تحتانی، ناحیه مرکزی (شکنج پیش‌مرکزی و پس‌مرکزی) و بخش قدامی شکنج گیجگاهی دچار ضایعه شده بود. در بیماران زبان‌پریش ورنیکه (8/48±66 ساله) نواحی کپسول خارجی- اینسولا، بخش خلفی شکنج گیجگاهی، بخش قدامی شکنج گیجگاهی و لوبول آهیانه‌ای تحتانی ضایعه دیده بود. نتیجه‌گیری: با توجه به جایگاه ضایعات مغزی در بیماران پژوهش حاضر، در هیچ یک از بیماران زبان‌پریش بروکا و ورنیکه، ضایعات مغزی تنها به ناحیه بروکا و ورنیکه محدود نشده بود. البته با توجه به تعداد اندک بیماران زبان‌پریش شرکت‌کننده در پژوهش حاضر، تعمیم نتایج آن به سایر افراد دارای زبان‌پریشی بروکا و ورنیکه دشوار است.

Title: تأ‌ثیر مکالمه با تلفن همراه بر زمان واکنش رانندگان به محرک شنوایی: بررسی نظریه منابع چندگانه و منبع مرکزی توجه

http://rehabilitationj.uswr.ac.ir/article-1-791-fa.html

هدف: هدف این تحقیق، بررسی نظریه منابع چندگانه و منبع مرکزی توجه در اجرای تکلیف ثانویه صحبت با دو نوع تلفن همراه حین رانندگی بود. روش‌بررسی: با استفاده از نمونه‌گیری در دسترس، تعداد 25 شرکت کننده مرد انتخاب و زمان واکنش آنان به محرک شنوایی با استفاده از نرم افزار و سخت افزار زمان واکنش در سه حالت مختلف رانندگی، بدون مکالمه با تلفن همراه، مکالمه با تلفن همراه با دست و مکالمه با تلفن همراه از طریق دوگوشی (هدفون) ثبت شد. به‌منظور کنترل اثر ترتیب و آشنایی شرکت‌کنندگان با آزمونها، ترتیب شرایط مختلف رانندگی به‌طور منظم از یک شرکت‌کننده به شرکت کننده دیگر تغییر یافت. یافته‌ها: نتایج حاصل از تجزیه و تحلیل داده ها با روشهای آمار توصیفی و با استفاده از آزمون کرویت مخلی ، آنالیز واریانس تک عاملی با اندازه گیریهای مکرر و آزمون تی داده های همبسته نشان داد که شرایط مختلف رانندگی بر زمان واکنش تاثیر می گذارد (P<0/001). مکالمه با تلفن همراه دستی زمان واکنش ساده رانندگان به محرک شنوایی را افزایش داد (P>0/001). مکالمه با تلفن همراه هدفن دار زمان واکنش ساده رانندگان به محرک شنوایی را افزایش داد (P<0/001). همچنین، استفاده از تلفن همراه دستی زمان واکنش رانندگان به محرک شنوایی را بیش از تلفن همراه هدفن دار افزایش نداد (P<0/001). نتیجه‌گیری: نتایج بدست آمده مؤید این است که مکالمه با تلفن همراه، چه دستی، چه هدفن دار، زمان واکنش رانندگان را افزایش می دهد. اجرای تکالیف دوگانه و چندگانه با مدل چهار بعدی نظریه منابع چندگانه قابل پیش بینی است. پیشنهاد می شود که قوانین راهنمایی و رانندگی که در ارتباط با تلفن همراه دستی اعمال می شود به استفاده از تلفن همراه هدفن دار نیز تسرّی یابد.

Title: توسعه نسخه‌ فارسی پرسشنامه کیفیت زندگی سازمان جهانی بهداشت-۱۰۰

http://rehabilitationj.uswr.ac.ir/article-1-848-fa.html

هدف: این مطالعه، با هدف ترجمه پرسشنامه کیفیت زندگی سازمان جهانی بهداشت‌-۱۰۰ به زبان فارسی و ارزیابی روایی و پایایی نسخه ترجمه شده، انجام شد. روش‌بررسی: در این مطالعه استانداردسازی و اعتبارسنجی، از روش پیشروـ‌پسرو جهت ترجمه پرسشنامه استفاده شد. در مطالعه مقدماتی، از یک نمونه 60 نفری از دانشجویان برای ارزیابی تکرارپذیری و همچنین روایی هم‌زمان بهره گرفته شد. سپس، 500 فرد سالم و 500 فرد معلول به‌صورت چندمرحله‌ای، به‌تصادف انتخاب شدند. پایایی درونی نسخه فارسی پرسشنامه کیفیت زندگی سازمان جهانی بهداشت‌-‌۱۰۰ با استفاده از آلفای کرونباخ در این نمونه مورد ارزیابی قرار گرفت. علاوه بر این، برای سنجش روایی تشخیصی این ابزار، یک مدل رگرسیون خطی چندگانه به‌کار رفت. یافته‌ها: نتایج آزمون‌ـ‌بازآزمون مطالعه مقدماتی نشان داد که نسخه فارسی پرسشنامه کیفیت زندگی سازمان جهانی بهداشت‌-‌۱۰۰ قابلیت تکرارپذیری قابل‌قبولی دارد (ضرایب هم‌بستگی درون‌گروهی تمامی شش بعد بالاتر از 0/7 بود). همچنین محاسبه ضرایب هم‌بستگی ابعاد مختلف دو پرسشنامه کیفیت زندگی سازمان جهانی بهداشت‌-۱۰۰ و کیفیت زندگی سازمان جهانی بهداشت‌ـ‌فرم کوتاه، روایی هم‌زمان رضایت‌بخشی را نشان داد. تحلیل داده‌های 500 فرد سالم و 500 فرد معلول آشکار ساخت که تمامی ابعاد پرسشنامه کیفیت زندگی سازمان جهانی بهداشت‌-‌۱۰۰ ترجمه‌‌شده، حداقل مقدار قابل‌قبول برای پایایی درونی را احراز نموده‌اند. نتایج تحلیل رگرسیون نیز نشان‌دهنده روایی تشخیص قابل پذیرشی برای تمامی ابعاد این ابزار به غیر از بعد معنوی بود. نتیجه‌گیری: نسخه فارسی پرسشنامه کیفیت زندگی سازمان جهانی بهداشت‌-‌۱۰۰ روایی و پایایی رضایت‌بخشی برای ارزیابی کیفیت زندگی فارسی‌زبانان دارد. تحقیقات بیشتر در خصوص بعد معنوی توصیه می‌شود.

Title: بررسی فرهنگ سازمانی موجود و مطلوب از دیدگاه کارکنان مراکز روزانه سازمان بهزیستی شهر تهران

http://rehabilitationj.uswr.ac.ir/article-1-849-fa.html

هدف: هدف از طرح و اجرای این تحقیق ارزیابی سه روش متداول ورزش درمانی در بیماران مبتلا به کمر درد و مطالعه چگونگی تاثیر هریک از این ورزش ها بر شدت درد و در صد ناتئانین ناشی از کمر درد است. روش بررسی: این مطالعه به صورت کارآزمایی بالینی بر روی 25 بیمار مبتلا به کمر درد مزمن مکانیکی با شرایط شغلی یکسان انجام شد. میزان شدت درد توسط جدول ارزیابی عددی درد ، و درصد ناتوانی ناشی از کمردرد توسط پرسشنامه اوسوستری مورد ارزیابی قرار گرفت. میزان اعتبار داده ها نیز بوسیله آزمون کندال محاسبه شد. افراد مورد تحقیق به سه زیر گروه ه تقسیم شدن به گونه ای که میانگین متغیرها در سه گروه، اختلاف معننی داری با یکدیگر نداشتند. گروه اول با تعداد 8نفر ورزش های ویلیامز را انجام دادند و گروه دوم با تعداد 7 نفر ورزش های مکنزی، و گروه سوم با تعداد 10 نفر بودند که ورزش های ثبات دهنده را انجام  دادند. طبق برنامه هر گروه طی دو مرحله 6 هفته ای (6 هفته اول 6 روز در هفته و در 6 هفته دوم 3 روز در هفته-یکروز در میان) و در مجموع آزمون های نویلکاکسون – فریدمن – کروسکال والیس و منویتنی انجام شد. یافته ها: نتایج به دست آمده نشان دادند که ورزش های انجام شده در هر سه گروه با کاربرد وزش های تقیتی عضلات ستون فقرات می توانند بر کاهش درد بیماران موثر باشند. البته زمان و سرعت کاهش درد، درصد ناتوانی و ماندگاری تاثر ضد درد در گروهی که ورزش های ثبات دهنده را انجام داد با دو گروه دیگر اختلاف معنی دار وجود داشت(p=0.0I) نتیجه گیری: بنابراین در هر سه روش درمانی درد بیمار کاهش می یابد، لیکن ورزش های ویلیامز و مکنزی  همیشه در شرایط استاتیک انجام می شوند و یک گروه عضلانی خاص را تقیت می کنند، اما در ورزش های ثبات دهند، علاوه تقویت عضلات در شرایط استاتیک تقویت عضلات، در شرایط دینامیک نیز صورت می گیرد. بیمار از شرایط انجام تمرینات سبک به سمت تمرینات سنگین، و نهایت به سوی بهبود شرایط تعادل از دست رفته پیش می رود، به همین دلیل انجام این ورزش ها علاوه بر تسریع در کاهش درد موجب بهبود شرایط ذهنی فرد، بهبود اعتماد به نفس و در نهایت کاهش درصدر ناتوانی ناشی از کمردرد می شود.
